# Museum of the Atlanta Medical College

**Published:** 1858-08

**Authors:** 


### Museum of the Atlanta Medical College.

The room appropriated to the Museum in the Atlanta Medical College having been completed, we would announce to our friends that it is now ready for the reception of whatever they may be disposed to contribute.

Through the kindness of our medical friends, in various directions, (in addition to purchases made by the Faculty,) we are now enabled to present quite an interesting and respectable nucleus, which will, we are sure, rapidly increase under the efficient management of the recently appointed Curator, Dr. N. D'Alvigny.

There are hundreds of interesting specimens in the possession of the medical men of Georgia alone, which are at present of but little comparative use, but collected in a well arranged Museum would become profitable to many. We, therefore, earnestly request medical men not only in our own State, but in others, to aid us in this way, and transmit whatever they may have, possessing the slightest interest, with its history, to the address of N. D'Alvigny, M. D., Curator, Atlanta Medical College, Atlanta, Geo.

Of course all expenses of transportation will be paid by the College. In all cases, the specimens will be labelled with the name of the donor, the history of which will be preserved in a descriptive catalogne.

